# Supplementary material for: Sugarcane Rhizosphere Bacteria Community Migration Correlates with Growth Stages and Soil Nutrient
Source: Int J Mol Sci. 2022 Sep 7;23(18):10303. doi: 10.3390/ijms231810303 (PMC9499485; doi:10.3390/ijms231810303)
Supplement: Supplementary file 1 [file ijms-23-10303-s001.zip › ijms-1889446-supplementary.pdf]

International Journal of *Molecular Sciences*

MDPI

Table S1. Soil physicochemical properties of each season.

Table S1 Soil physicochemical properties of each season				
	May	July	Sep	Nov
pH	6.22±0.04 a	5.83±0.05 b	5.55±0.06 c	5.38±0.09 c
OM	26.78±0.89 b	25.03±0.54 b	30.80±1.91 a	26.71±1.19 b
TN	2.74±0.04 a	2.45±0.02 b	2.39±0.03 b	2.40±0.02 b
TP	0.80±0.01 b	0.78±0.01 b	0.92±0.02 a	0.82±0.01 b
TK	23.25±0.20 a	16.58±0.44 c	19.65±0.24 b	17.18±0.28 c
AN	182.06±5.73 a	168.75±3.12 b	127.01±1.67 c	125.04±3.46 c
AP	78.61±1.87 ab	82.08±1.19 a	65.47±1.48 c	75.46±2.39 b
AK	155.99±4.08 a	149.97±3.49 a	136.11±5.70 ab	125.11±14.72 b

Note: Different letters in each column indicate significant difference among the treatments at 0.05 level.

Table S2 Topological properties of bacterial interaction network in rhizosphere soil of sugarcane at different growth stages				
	May (Seeding)	July (Tillering)	Sep (Elongation)	Nov (Maturity)
Number of nodes	1099	1705	1276	1128
Number of edges	5922	29822	16005	62237
Network density	0.01	0.021	0.015	0.098
Modularity	0.76	0.846	0.869	1.707
Clustering coefficient	0.303	0.348	0.347	0.546
path length	4.144	3.436	3.535	2.9
Positive	83.06%	70.99%	71.91%	57.64%
Negative	16.94%	29.01%	28.09%	42.36%

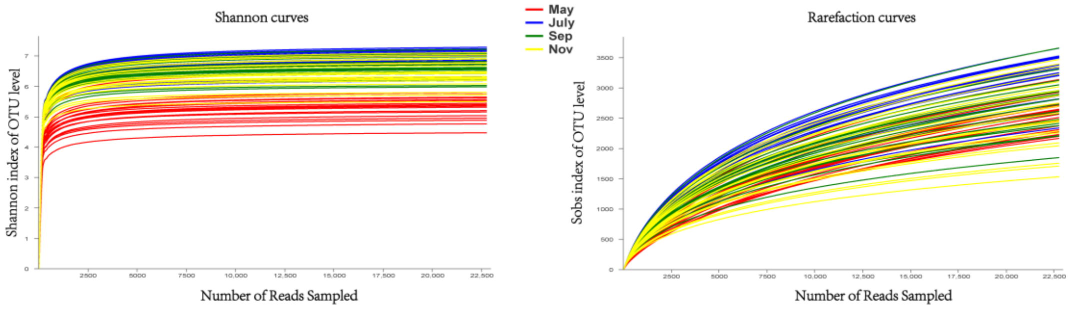

Figure S1. The rarefaction curves of bacteria. The rarefaction curves are drawn by randomly selecting a certain number of sequences from the sample, counting the Alpha diversity index of these sequences corresponding to the sample, plotting the curve with the amount of data extracted as the horizontal coordinate and the Alpha diversity index value as the vertical coordinate, and the curve gradually approaches flatness indicating sufficient amount of sequencing data. Different colors in the picture represent different seasons.

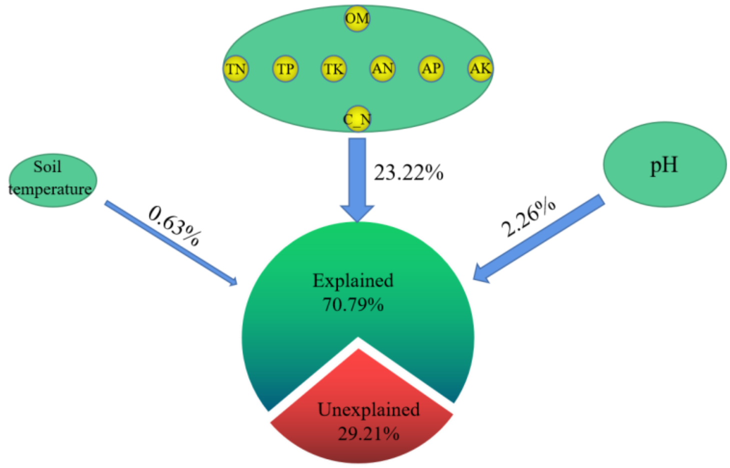

Figure S2. Variation partitioning analysis (VPA) of the effects of soil edaphic factors on bacteria community structure. Green represents the proportion of changes in bacteria community structure induced by soil properties, red represents the proportion of unexplained.

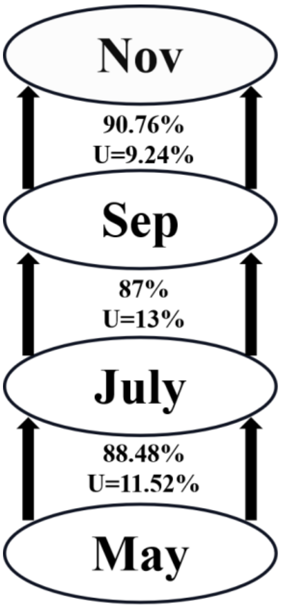

Figure S3. Source Model of Plant Microbiome (SMPM) depicting the potential sources of bacteria community structure in 84 samples of sugarcane rhizosphere soil. "U" represents the proportion of bacteria from unknown source.

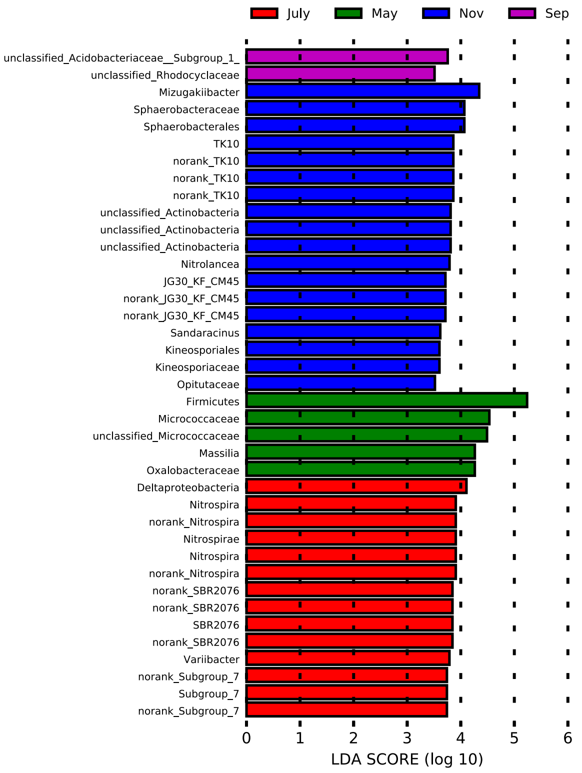

Figure S4. Liner discriminant analysis (LDA) coupled with effect size measurements identifies the differentially abundant taxa between different seasons, May (Green), July (red), September (purple), and November (blue). Lineages with LDA values higher than 2.5 are displayed.

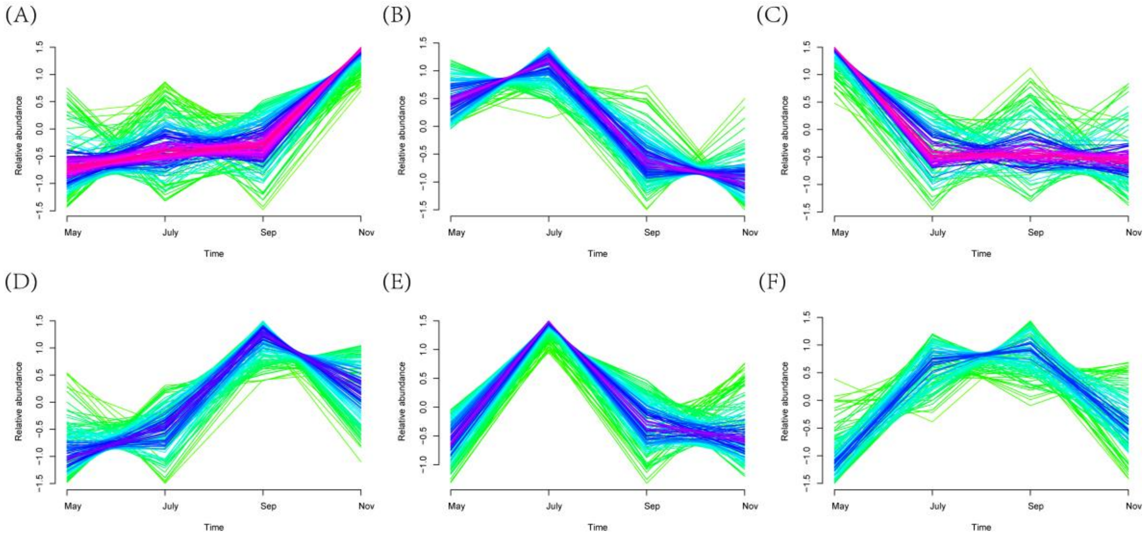

Figure S5. Based on all rhizosphere bacterial genera, clustering results of Cluster (A) 1, (B) 2, (C) 3, (D) 4, (E)5 and (F)6. Red shades indicate high membership values and green shades low membership values of bacterial.

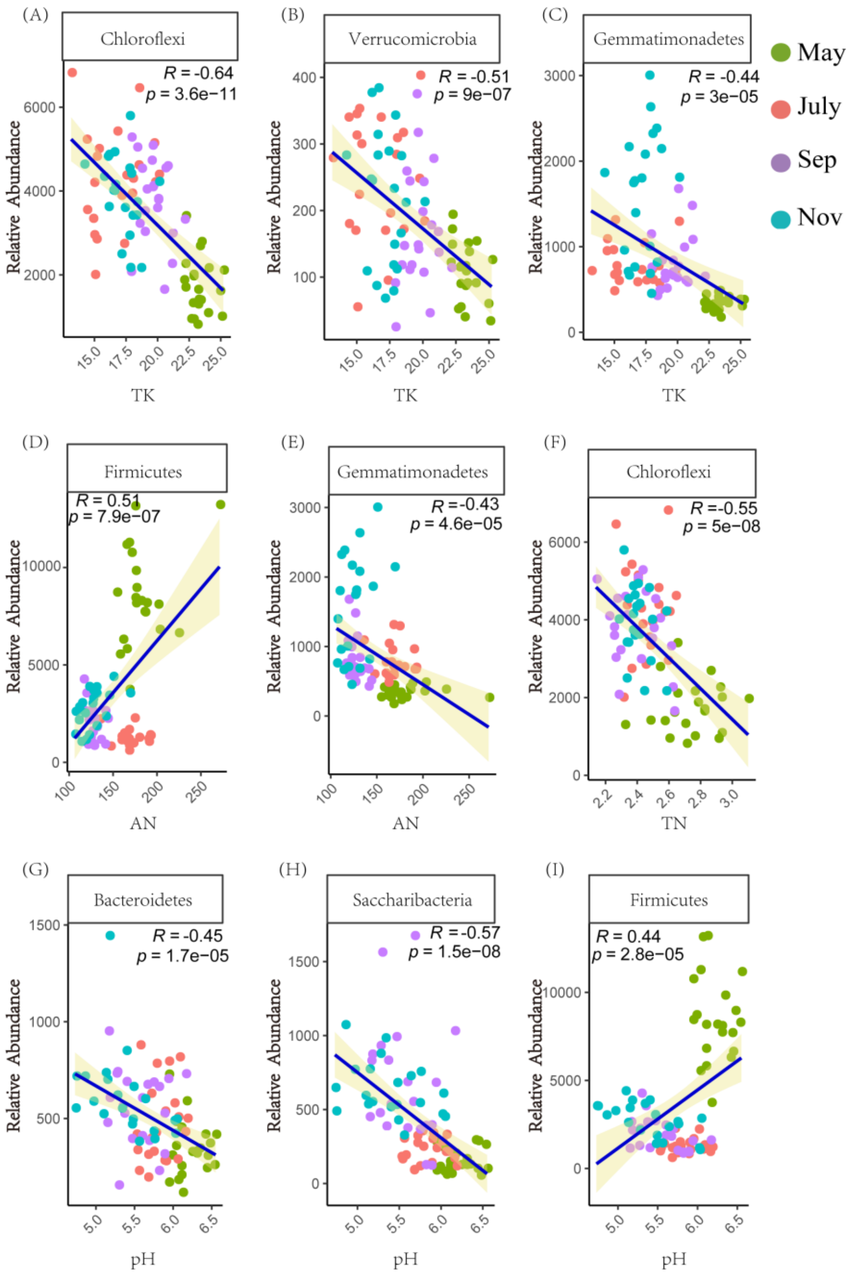

Figure S6. (A-I) Linear relationships among relative abundance of selected bacterial phyla and soil physicochemical variables ($p < 0.05$). The ordinate is relative abundance, and the abscissa is soil properties.
